# Geographical equity in Ethiopian infant feeding practices: a secondary analysis of a birth cohort study

**DOI:** 10.1136/bmjopen-2024-088762

**Published:** 2025-01-02

**Authors:** Amare Tariku, Lars Persson, Joanna Schellenberg, Tanya Marchant, Della Berhanu, Seblewengel Lemma, Atkure Defar, Theodros Getachew Zemedu, Zewditu Abdissa Denu, Tadesse Guadu Delele, Solomon Shiferaw, Girum Taye Zeleke, Meseret Zelalem, Kassahun Alemu Gelaye

**Affiliations:** 1 Institute of Public Health, College of Medicine and Health Sciences, University of Gondar, Gondar, Ethiopia; 2 Department of Disease Control, London School of Hygiene & Tropical Medicine, London, UK; 3 Health System and Reproductive Health Research Directorate, Ethiopian Public Health Institute, Addis Ababa, Ethiopia; 4 School of Public Health, Addis Ababa University, Addis Ababa, Ethiopia; 5 Maternal, Child & Adolescent Health Service Lead Executive Office, Ethiopia Ministry of Health, Addis Ababa, Ethiopia

**Keywords:** NUTRITION & DIETETICS, PUBLIC HEALTH, Child

## Abstract

**ABSTRACT:**

**Objectives:**

To assess the geographical equity in Ethiopian infants’ exclusive breastfeeding at 5 months and dietary diversity at 12 months and whether social factors explained the spatial inequities.

**Design:**

Secondary analysis of a birth cohort study.

**Setting:**

Analysis of data from the Ethiopian Performance Monitoring for Action panel study conducted from July 2020 to August 2021 in five regions (ie, Oromia, Amhara, Afar and Southern Nations, Nationalities and Peoples regions and the Addis Ababa City administration). We analysed geographical autocorrelation to assess geographical variations in exclusive breastfeeding and dietary diversity. Areas with higher and lower coverage of appropriate infant feeding outcomes were analysed using hotspot analyses. We performed geographically weighted regression to investigate whether sociodemographic factors explained the geographical feeding differences.

**Participants:**

1850 infants were prospectively followed from birth to 12 months.

**Outcome measures:**

Exclusive breastfeeding at 5 months and minimum-level dietary diversity at 12 months.

**Results:**

Sixty-nine percent (95% CI 67 to 71) of infants were exclusively breastfed at 5 months, while at 12 months, only 16% (95% CI 13 to 19) had complementary feeding from five or more food groups. There were geographical variations in the coverage of exclusive breastfeeding and minimum dietary diversity. Higher proportions of infants were exclusively breastfed at 5 months in northern areas, and more 12-month-old infants in central parts of the country had complementary feeding from at least five food groups. Sociodemographic factors explained a quarter of the geographical variation in dietary diversity.

**Conclusion:**

Most Ethiopian infants were exclusively breastfed, which was in line with global recommendations but with some geographical differences. There was prominent geographical variation in dietary diversity, partly explained by social factors, but overall, very few 12-month-old infants were fed from at least five food groups. This low dietary quality could impair Ethiopian children’s physical growth, development and health.

**STRENGTHS AND LIMITATIONS OF THIS STUDY**

- This Ethiopian birth cohort study is based on a nationally representative sample and provides insight into the geographical variation in exclusive breastfeeding of 5-month-old infants and their dietary diversity at 12 months.
- Mother-infant pairs followed from birth to the first birthday were selected from five administrative regions, representing about 85% of the Ethiopian population.
- Exclusive breastfeeding was prospectively measured, minimising recall bias.
- Dietary diversity was based on 24-hour recall, which enabled mothers to report infants’ dietary intake and provide valid group estimates but limited the individual-level assessment of overall dietary habits.

## Introduction

 The Sustainable Development Goals (SDGs) included targets to end child undernutrition and reduce under-five mortality to less than 25 per 1000 live births.^1^ The WHO and UNICEF recommend appropriate infant feeding as a key strategy to tackle child growth faltering and enhance child survival.^2^ Narrowing geographical infant feeding inequities is essential to enhancing appropriate breastfeeding and complementary feeding practices.^3 4^

The WHO and UNICEF recommend that all newborns be put to the breast within 1 hour after birth.^2^ Although almost all (98%) infants in low- and middle-income countries breastfeed at some point in time,^5^ estimates from 2010 to 2028 suggest that only half of newborns were put to the breast within 1 hour after birth.^6^ Studies have shown regional variations in the coverage of early breastfeeding initiation. The lowest coverage was found in Southeast Asia (47%) and the highest in European regions (67%). Half of the infants in African or sub-Saharan African countries benefited from early breastfeeding initiation. Despite the recommendation that all infants exclusively feed on breast milk up to 6 months of age,^2^ less than half (48%) in low- and middle-income countries were exclusively breastfed.^7^ Studies showed significant geographical variation in exclusive breastfeeding practices across the WHO regions.^6^ The lowest prevalence of exclusive breastfeeding was found in the Eastern Mediterranean region (35%), and the highest was in Southeast Asia and the Western Pacific region (55%). Most of the low- and middle-income countries in South America and South and East Asia had a high coverage of exclusive breastfeeding.^3^ Two-thirds of low- and middle-income countries are unlikely to reach the 2030 target of exclusive breastfeeding, which is 70% or more being exclusively breastfed at 5 months.^7^ In 2018, less than half (42%) of African infants below 6 months were exclusively breastfed.^6^ There were major geographical differences in exclusive breastfeeding in Africa, with more than twofold differences in the prevalence of exclusive breastfeeding between districts, and a low chance of reaching the exclusive breastfeeding target.^8^

Ethiopia is committed to ensuring optimal child nutrition and enhancing child survival by implementing infant and young child nutrition strategies.^1^ Despite important improvements during the Millennium Development Goal era,^9^ child mortality remains high (59 per 1000 live births) with geographical heterogeneity.^10 11^ We have reported elsewhere that more than two-thirds of Ethiopian infants initiated breastfeeding within 1 hour after birth and had exclusive breastfeeding at 5 months of age, while very few had complementary feeding comprising five or more food groups at 12 months.^12^ Mapping disparities in infants’ breastfeeding and complementary feeding practices are essential to inform child survival efforts by ensuring optimal access to infant feeding support.^3^ Few national studies have examined the geographical variation in early initiation of breastfeeding^13^ and vitamin-rich food intake (6–23 months).^14^ There is a scarcity of analysis of geographical variation in infants’ exclusive breastfeeding and dietary diversity of complementary feeding. In this birth cohort study, we investigated the geographical variation in infants’ exclusive breastfeeding at 5 months of age and the coverage of minimum-level diet diversity at 12 months. We also analysed to what extent socio-demographic factors explained the geographical variation in exclusive breastfeeding and the dietary diversity of complementary feeding.

## Methods

### Study design and setting

This analysis was based on birth cohort data from the Performance Monitoring for Action (PMA) study conducted from July 2020 to August 2021 in five Ethiopian regions, that is, the large and predominantly agrarian regions Oromia, Amhara and Southern Nations, Nationalities and Peoples; the urban Addis Ababa; and pastoralist Afar region.^15^ Our study did not include data from Tigray as fieldwork was discontinued in that region in November 2020 because of armed conflict. The five regions included 85% of the Ethiopian population. The PMA was a collaborative project between Addis Ababa and Johns Hopkins Universities and evaluated the coverage, quality and comprehensiveness of the continuum of Reproductive, Maternal and Newborn Health services, including a birth cohort follow-up to 12 months.

### Study population and sampling

This birth cohort study included all pregnant women and those within 6 weeks post partum, aged 15–49 years, who were regular household members or resided with parents for pregnancy or postpartum care. The selection was based on a multistage cluster sampling to invite women fulfilling the eligibility criteria. The sampling frame was generated using a list of clusters (enumeration areas (EAs)) obtained from the Ethiopian Central Statistical Agency.^16^ The EAs were selected with probability proportional to the size of the study regions, and our analysis was based on 183 EAs. All pregnant or postpartum women within 6 weeks after birth aged 15–49 years who lived in the study clusters and consented to participate were included.

### Data collection

The panel data were collected using questionnaires jointly developed by the PMA research team. Questionnaires were piloted before data collection. The field teams used interview guides to ensure data quality. The study recruited regional coordinators, field supervisors and data collectors from the local areas. Two weeks of training supported by 3 days of field exercises were given to regional coordinators, field supervisors and data collectors. Three follow-up interviews were conducted. The first was conducted about 6 weeks after delivery, the second at 6 months and the third when the infants were 12 months old. The central data management team sent automated reminder text messages to data collectors a week before the scheduled interviews. Details about the sampling, design and data collection are published elsewhere.^15^

### Outcome measures

The study’s primary outcomes were exclusive breastfeeding at 5 months of age and dietary diversity at 12 months. An infant was considered to have early initiation of breastfeeding if they were put to the breast within 1 hour after birth.^2^ At the follow-up interviews, mothers were asked if and at what age the infant was introduced to any other food or fluid besides breast milk. Infants were considered to have exclusive breastfeeding when they only received breast milk. At all interviews, mothers were asked whether infants were breastfed in the 24 hours before the interview. Infants’ complementary feeding was assessed at the third follow-up, and mothers were asked about the food items given to the infant in the 24 hours before the interview. We have previously given more details about infants’ dietary intake at 12 months.^12^

### Analyses

We analysed participation and loss to follow-up among eligible women with live births. We displayed socio-demographic characteristics and infants’ breastfeeding and complementary feeding practices with descriptive statistics and 95% CIs. Our analyses were adjusted for sampling weights to ensure that the included women represented the Ethiopian population. Sampling weights were calculated as the inverse probability of the enumeration area (primary sampling unit) and household (secondary sampling unit) selections. A principal component analysis was employed to represent the household socio-economic status with a wealth index based on the ownership of livestock, assets and house materials. The household wealth index was divided into quintiles from the poorest to the least poor.

Early breastfeeding initiation was shown as the percentage of newborns put to the breast within 1 hour after birth. The coverage of exclusive breastfeeding was calculated as the proportion of infants who were fed only breast milk at 5 months. Infants were reported to have continued breastfeeding if they were fed breast milk within the 24 hours before the interview. The coverage of at least minimum diet diversity was estimated as the proportion of infants aged 12 months given complementary food from five or more food groups.

During the household census, the location of households was geocoded using global positioning system (GPS) coordinates (degrees in latitude and longitude). The actual location of the households was concealed for privacy reasons. The PMA used the same geographical location displacement protocol as the Ethiopian Demographic and Health Survey, where an EA GPS centroid was randomly displaced in a direction ranging from 0 to 360°. Due to the variation in population density, the urban geographical displacement was 2 km and the rural 5 km.

ArcGIS V.10.3 software was used to analyse whether appropriate breastfeeding and complementary feeding were clustered, dispersed or randomly distributed across the Ethiopian regions. The central data management team used QGIS V.3.14 to regularly check whether interviews were conducted within the study area by reviewing the distance between each household and the centre of the EA.^15^ The spatial autocorrelation of infant feeding practices was examined using Global Moran’s I spatial statistic. The Global Moran’s I value of ‘0’ showed a random distribution of feeding practices, while −1 and +1 indicated feeding practices dispersed or clustered, respectively. The statistical significance of geographical clustering was considered when a p value was <0.05.^17^ A hotspot analysis using Getis-Ord Gi* statistics was employed to illustrate areas with lower (cold spot) or higher (hotspot) prevalence of exclusive breastfeeding and at least minimum diet diversity.^18^ We did an ordinary kriging Gaussian interpolation analysis to predict the prevalence of exclusive breastfeeding and minimum diet diversity across the country based on values taken from the study areas.

We performed an ordinary least-square analysis to examine whether selected sociodemographic characteristics explained part of the geographical variation in coverage of exclusive breastfeeding at 5 months and minimum diet diversity. We examined the Koenker BP and Jarque-Bera statistics to see if the stationarity assumption was violated (supported by p value <0.05), that is, socio-demographic characteristics explaining the geographical variation in infant feeding. Koenker BP and Jarque-Bera statistics guided the geographically weighted regression (GWR) analysis so that independent variables with a p value <0.05 were selected for the analysis.^19^ A variance inflation factor greater than 10 indicated multicollinearity among the explanatory variables. A p value <0.05 was considered statistically significant in the GWR analysis. We used the latest (2013) Ethiopian geographical shapefile for all geographical analyses.

### Patient and public involvement

None.

## Results

### Participation

In the larger PMA study, 27 722 women of reproductive age were screened for eligibility. 2585 were found pregnant or within 6 weeks post partum, that is, fulfilled the eligibility criteria. The first follow-up interview was conducted among 2230 mothers with 2304 live births. Mothers of 1850 infants (80.3%) completed the three follow-up interviews up to 12 months, while 454 were lost to follow-up mainly because of pregnancy losses, absence at the time of interview or incomplete data.

### Socio-demographic characteristics

Three-fourths of the mothers were rural residents, and 17% had attended secondary school or above (table 1). One-tenth of the mothers had four or more antenatal care visits, and 55% gave birth at health facilities. Only one-tenth and one-fifth of the mothers had received counselling on exclusive breastfeeding and complementary feeding, respectively. Details about the socio-demographic characteristics of the mothers and a study flow diagram have been published elsewhere.^12^

**Table 1:** Sociodemographic characteristics of mothers of infants who completed 12 months of follow-up, PMA Ethiopia panel study, July 2020 to August 2021 (n=1850)

Characteristics	Unweighted N (%)	Weighted* % (95% CI)
Region		
Afar	178 (10)	2 (1 to 3)
Amhara	413 (22)	24 (21 to 27)
Oromia	539 (29)	46 (41 to 51)
Southern Nations, Nationalities, and Peoples	506 (27)	24 (20 to 28)
Addis Ababa	214 (12)	4 (3 to 5)
Residence		
Urban	640 (35)	21 (16 to 27)
Rural	1210 (65)	79 (73 to 84)
Household wealth quintiles (n=1849)		
1 (poorest)	353 (19)	21 (16 to 27)
2	297 (16)	20 (17 to 23)
3	312 (17)	21(18 to 24)
4	331 (18)	19 (16 to 24)
5 (Least poor)	556 (30)	19 (14 to 24)
Mothers’ age in years (n=1849)		
15–19	145 (8)	9 (7 to 10)
20–24	433 (23)	23 (21 to 26)
25–29	624 (34)	33 (30 to 35)
30–34	364 (20)	20 (17 to 22)
35–47	283 (15)	17 (15 to 19)
Education (n=1849)		
No schooling	753 (41)	43 (38 to 48)
Primary school	671 (36)	40 (36 to 44)
Secondary school or above	425 (23)	17 (14 to 21)
Number of antenatal care visits (n=1848)		
0	487 (26)	23 (19 to 29)
1	122 (7)	9 (6 to 12)
2	199 (11)	13 (11 to 16)
3	348 (19)	22 (19 to 25)
≥4	691 (37)	33 (28 to 38)
Place of delivery (n=1849)		
Health facility	1097 (59)	55 (49 to 61)
Home	752 (41)	45 (39 to 51)

* Analysis adjusted for enumeration area and household sampling weights.

PMAPerformance Monitoring for Action

### Feeding practice of infants aged 0–12 months

Sixty-seven percent initiated breastfeeding within 1 hour after birth and an additional 26% were put to the breast after 1 hour but within a day. All infants commenced breastfeeding during the first week of life. At 5 months, 69% were exclusively breastfed. The median duration of exclusive breastfeeding was 6.5 months (IQR 1 month). Ninety-seven percent were continuing breastfeeding at 12 months. Infants’ complementary feeding at 12 months was primarily based on grains, roots and tubers. Two-fifths of infants had complementary food based on pulses and nuts (43%) or dairy products (45%). Very few (6%) consumed iron-rich foods. Only 16% of infants had a mix of complementary food meeting the minimum diet diversity, that is, food from five or more food groups.

### Geographical distribution of exclusive breastfeeding at 5 months

**Figure 1:**
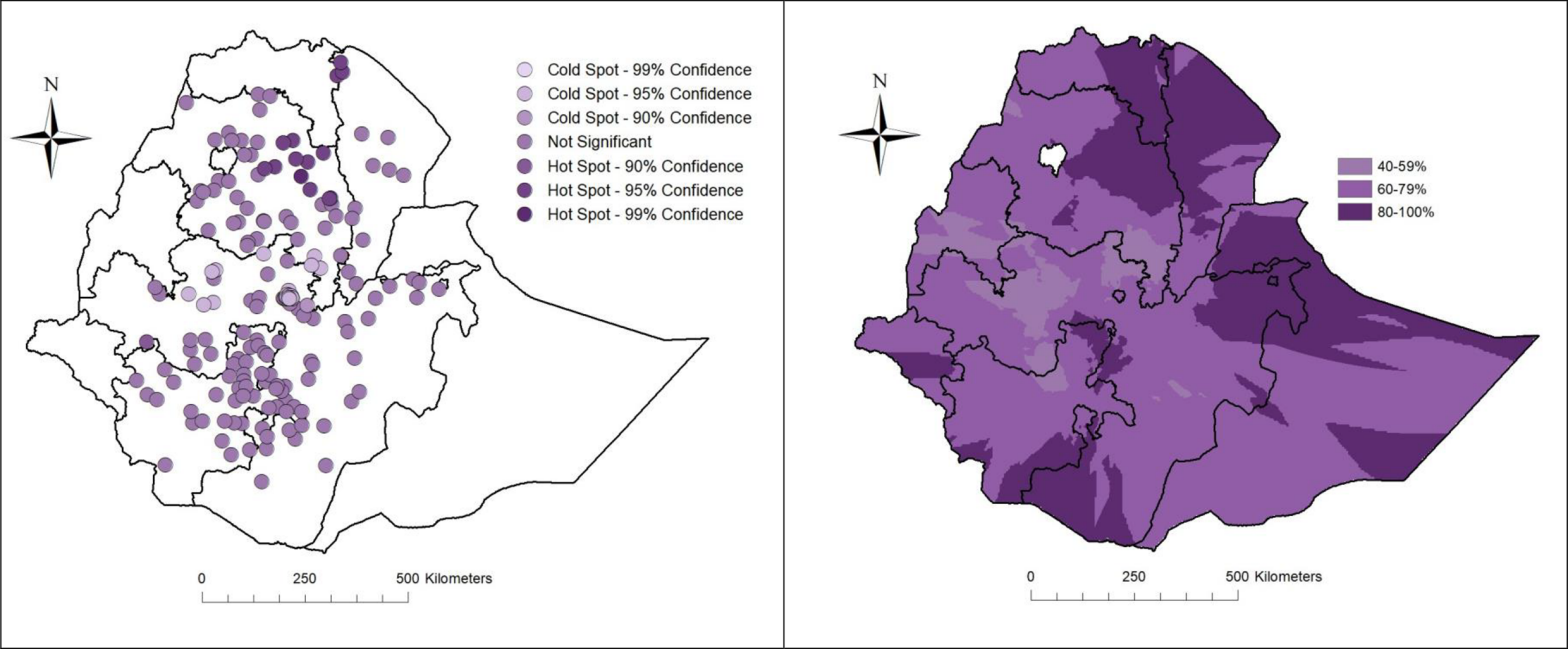
Hotspot analysis and kriging interpolation of infants’ exclusive breastfeeding at 5 months of age in Ethiopia, July 2020 to August 2021 (n=1850).

There was a significant geographical variation of exclusive breastfeeding at 5 months of age (Global Moran’s I 0.086, p<0.001). The highest coverage of exclusive breastfeeding was displayed in the northern part of Ethiopia, while the lowest was shown in Central Ethiopia (figure 1). The lowest and highest predicted prevalence of exclusive breastfeeding ranged from 41% to 100%.

### Geographical distribution of infants’ dietary diversity at 12 months

**Figure 2:**
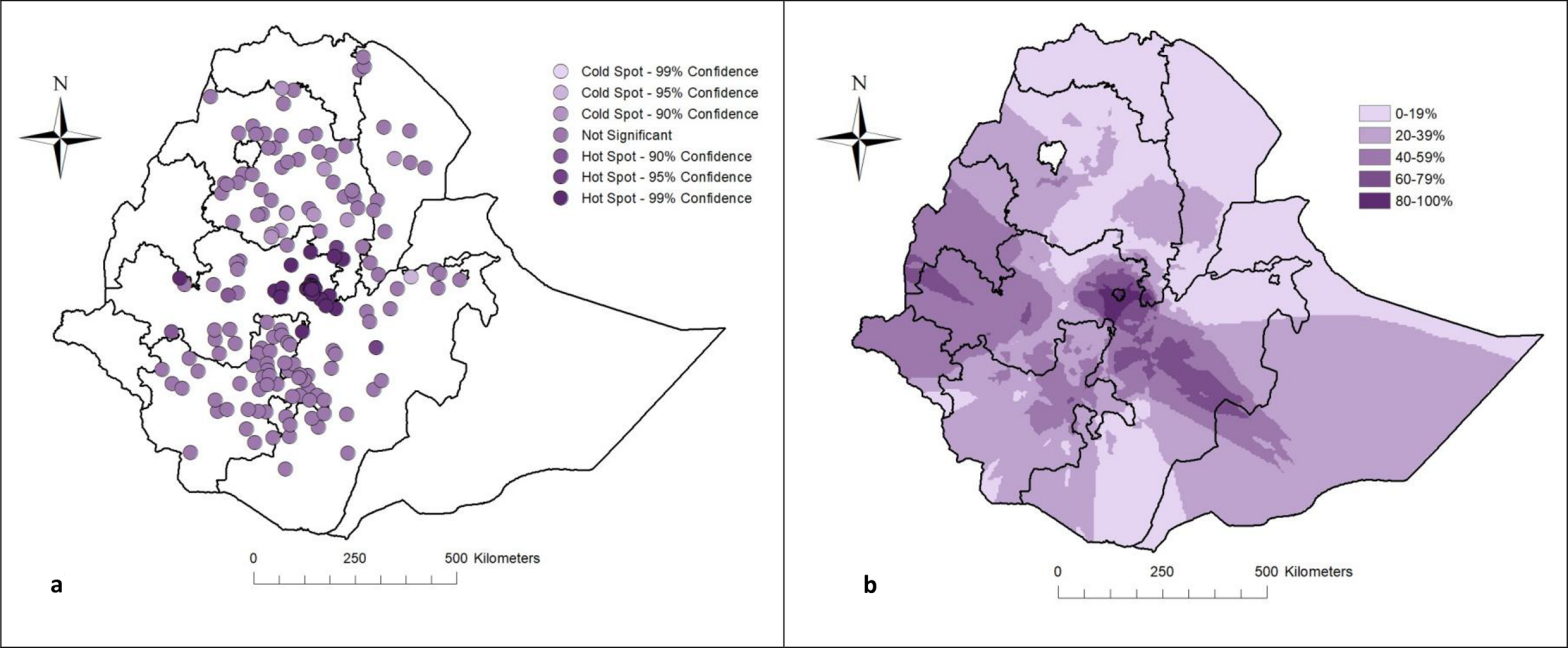
Hotspot analysis (**a**) and kriging interpolation (**b**) of minimum diet diversity among infants aged 12 months in Ethiopia, July 2020 to August 2021 (n=1850).

There was a significant geographical variation in the coverage of at least minimum-level dietary diversity (Global Moran’s I 0.12, p<0.001). In the central parts of the country, 40–59% of infants had complementary food meeting the dietary diversity criteria (figure 2a, b). Conversely, in the north, the coverage ranged from 0% to 19% and was the lowest in the country.

### Social determinants of the geographical variation in infant feeding

The mothers’ socio-economic characteristics and reproductive healthcare utilisation did not explain the geographical variation in the prevalence of exclusive breastfeeding at 5 months of age (online supplemental table 1). Household wealth and mothers’ education explained 28% of the geographical variation in the minimum-level dietary diversity (table 2). Mothers’ secondary or above education and the highest household wealth quintile explained the higher prevalence of minimum dietary diversity in the central parts of the country (online supplemental figure 1a,b).

**Table 2:** Summary of model diagnostic results for OLS and GWR analyses of determinants of geographical variation in the distribution of infants’ minimum dietary diversity, PMA Ethiopia panel study, July 2020 to August 2021 (n=1850)

Variable	Coefficient	Robust SE	Robust probability	VIF	Number of observations
Intercept	0.077	0.026	0.004	-	183
Wealthiest quintile	0.261	0.094	0.006	6.55	
Urban residence	−0.131	0.070	0.063	4.68	
Secondary or above education	0.287	0.089	0.001	2.73	
Model diagnostics					
Joint F-statistic	18.38				
Joint Wald statistic	65.229				
Koenker (BP) statistic	2.893				
Jarque-Bera statistic	202.311				
Akaike’s information criterion for OLS	−41.521				
Adjusted R^2^ for OLS	0.278				
Akaike’s information criterion for GWR	−40.736				
Adjusted R^2^ GWR	0.279				

GWRgeographical weighed regressionOLSordinary least squarePMAPerformance Monitoring for ActionVIFvariance inflation factor

## Discussion

Exclusive breastfeeding at 5 months met the global recommendation of 70% or more. Despite the relatively high coverage, there were some geographical differences, and these were not explained by elected sociodemographic characteristics. Only a tiny proportion of Ethiopian 12-month-old infants had complementary feeding with at least a minimum level of dietary diversity, that is, from at least five food groups. The sociodemographic characteristics of households and mothers partly explain the marked geographical variation in dietary diversity.

This study provided insights into the geographical equity of exclusive breastfeeding and dietary diversity in Ethiopian infants. The analysis was based on infants selected from five regions representing 85% of the country’s population. Exclusive breastfeeding was prospectively measured. The design generated more accurate estimates with less recall bias than in national surveys with recall periods of several years. Use of data collectors who resided in the community increased their familiarity with mothers and the study context. The use of local interviewers may have reduced the risk of social desirability or ‘White coat’ bias. Most of the eligible mothers (80.3%) completed the 1-year postpartum follow-up. The lost follow-up did not affect the coverage of exclusive breastfeeding at 5 months and minimum dietary diversity at 12 months of age. However, it might overestimate infants’ vitamin A and animal protein-rich (flesh, dairy and egg) food intake. Infants’ complementary food intake was assessed by 24-hour recall, a dietary assessment method with limited recall bias that generates appropriate estimates on the group level, although with the limitation of not showing the individual-level usual dietary habits.^20^

Our study showed that almost 70% of Ethiopian infants were exclusively breastfed at 5 months. This coverage implies that Ethiopia has reached the 2030 global exclusive breastfeeding target.^3^ This proportion was higher than earlier reported coverage of exclusive breastfeeding in low- and middle-income countries and sub-Saharan African countries.^6 8^ Although on a relatively high level, there were geographical variations in infants’ exclusive breastfeeding at 5 months of age. It was higher in the north and lower in Central Ethiopia. Geographical disparities in the coverage of exclusive breastfeeding have been reported from sub-Saharan Africa and other low- and middle-income countries.^3 8^ African and Asian studies have shown a considerably higher prevalence of exclusive breastfeeding in rural areas compared with urban settings.^21 22^ The lower prevalence of exclusive breastfeeding in Central Ethiopia, that is, the capital, Addis Ababa and the surrounding region, could be related to mothers’ high exposure to the marketing of breast milk substitutes.^23^ Logos of breast milk substitutes in Ethiopia were found posted in health facilities, and the nutritional labels of all breast milk substitutes violated at least one of the international codes of breast milk substitutes, including not mentioning the benefit and superiority of breastfeeding.^24^ Pervasive market promotion of breast milk substitutes with incorrect nutritional labelling targeting mothers and health workers was also reported in other low- and middle-income countries.^25^ Mass media, digital media and health facilities were the most typical marketing platforms, suggesting the critical importance of targeting these outlets in designing breastfeeding interventions. Integrated exclusive breastfeeding interventions in multiple settings, that is, mass media, workplace, community and health facilities, have been reported to have profound effects on exclusive breastfeeding practices.^26^ Because of the higher literacy rate, women in Addis Ababa are more likely to work outside their homes.^23^ Given 120 days of maternity leave, employed mothers might discontinue exclusive breastfeeding early. Lack of workplace breastfeeding support could also limit mothers’ ability to maintain exclusive breastfeeding.^23 27^

Only 16% of Ethiopian infants had complementary foods that met the minimum quality criteria. This proportion was lower than the prevalence reported from other low- and middle-income countries.^4^ The poor quality of complementary food suggests that infants are at risk of inadequate energy, protein and micronutrient intake.^28 29^ One-fourth of Ethiopians needed humanitarian support in 2023.^30^ Infants of families with food insecurity have been reported with an inadequate mix of complementary foods.^31^ Reducing the amount and frequency of meals was the main coping strategy in case of food insecurity.^31 32^ The proportion of infants with at least the minimum dietary diversity was higher in the central parts of the country, including the capital city. The lower coverage of minimum dietary diversity was seen in the northern parts of the country. Geographical inequity in dietary diversity has also been reported from other low- and middle-income countries.^4 33^ Improved market access and higher purchasing power in urban settings could explain the observed geographical variation. In other studies, infants’ dietary diversity has been higher in households closer to food markets.^34 35^ We found that household wealth and mothers’ education level explained some of the geographical variation, especially in the central parts of the country. Educated mothers may have more access to child nutrition education and behavioural change interventions.^36^ As has also been shown in another study, infants of educated and better-off mothers were more likely to have complementary foods with at least the minimum dietary diversity.^34^

## Conclusions

The proportion of Ethiopian infants being exclusively breastfed at 5 months of age was in line with the global breastfeeding target. A tiny proportion of infants had complementary feeding meeting the minimum dietary diversity criterion. Infants’ exclusive breastfeeding and diversity of complementary food varied geographically across the country. There was a higher prevalence of exclusive breastfeeding in northern Ethiopia, and more infants were fed with minimum dietary diversity in the central part of the country. Socio-demographic characteristics explained a quarter of the geographical variation in dietary diversity but did not explain any of the geographical variation in exclusive breastfeeding. Strengthening the implementation of breastfeeding interventions in Central Ethiopia is crucial to improving exclusive breastfeeding practices. It is also essential to increase access to infant feeding counselling and education services to improve the quality of complementary food.

## supplementary material

10.1136/bmjopen-2024-088762 online supplemental file 1

10.1136/bmjopen-2024-088762 online supplemental file 2

## Data Availability

Data are available in a public, open access repository.
