# Supplementary material for: Geographical equity in Ethiopian infant feeding practices: a secondary analysis of a birth cohort study
Source: BMJ Open. 2025 Jan 2;15(1):e088762. doi: 10.1136/bmjopen-2024-088762 (PMC11749732; doi:10.1136/bmjopen-2024-088762)
Supplement: online supplemental file 1 [file bmjopen-15-1-s001.docx]

Supplementary Table 1 Summary of model diagnostic results for Ordinary Least Square analysis for spatial determinants exclusive breastfeeding, PMA Ethiopia panel study, July 2020 to August 2021 (N= 1,850)

| Variable | Coefficient | Robust standard error | Robust probability | VIF | Number of observations |
| --- | --- | --- | --- | --- | --- |
| Intercept | 0.705 | 0.026 | 0.004 | ----- | 183 |
| Wealthiest quintile | -0.117 | 0.103 | 0.258 | 6.532 |  |
| Urban residence | 0.093 | 0.071 | 0.194 | 4.688 |  |
| Secondary or above education | -0.082 | 0.119 | 0.490 | 2.730 |  |
| Model diagnostics | | | | | |
| Joint F-Statistic | 1.310 |  |  |  |  |
| Joint Wald Statistic | 4.789 |  |  |  |  |
| Koenker (BP) Statistic | 2.319 |  |  |  |  |
| Jarque-Bera Statistic | 15.446 |  |  |  |  |
| Akaike’s Information Criterion | 38.496 |  |  |  |  |
| Adjusted R-Squared | 0.005 |  |  |  |  |
