# Supplementary material for: Geographical equity in Ethiopian infant feeding practices: a secondary analysis of a birth cohort study
Source: BMJ Open. 2025 Jan 2;15(1):e088762. doi: 10.1136/bmjopen-2024-088762 (PMC11749732; doi:10.1136/bmjopen-2024-088762)
Supplement: online supplemental file 2 [file bmjopen-15-1-s002.pdf]

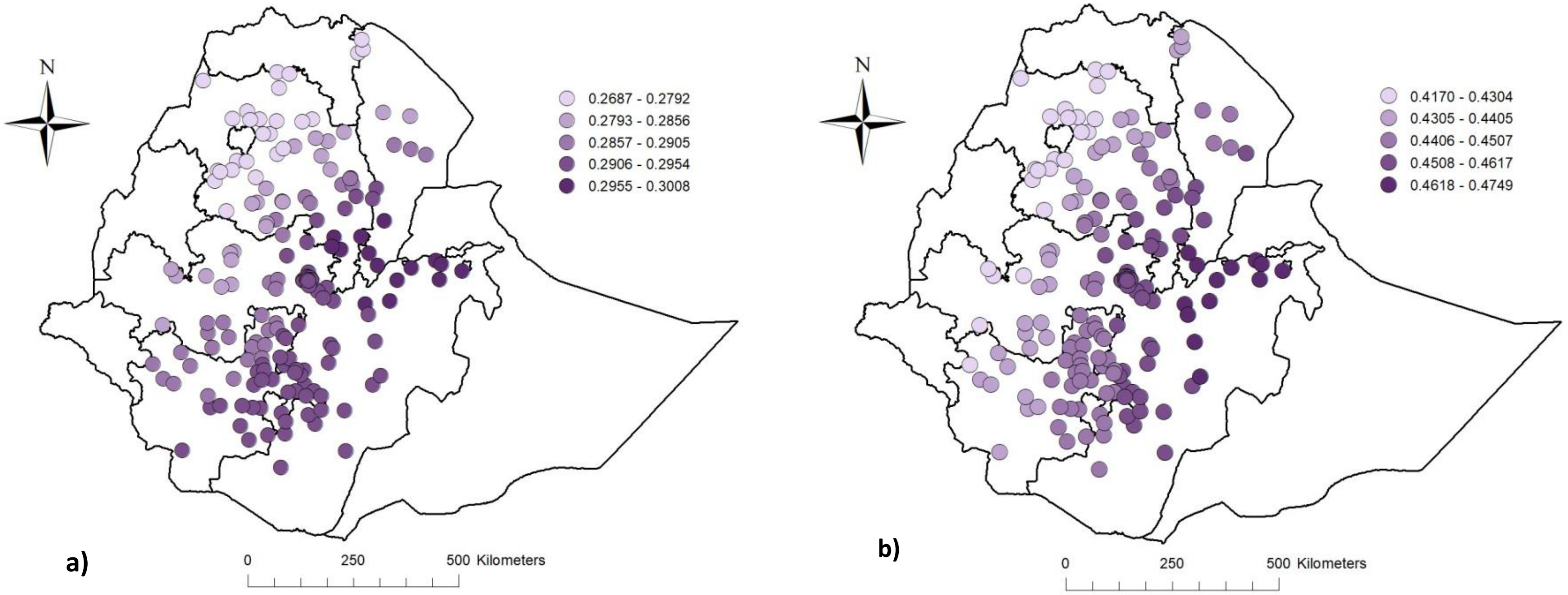

Supplementary Figure 1: Geographic weighted regression analyses for minimum dietary diversity by mothers' wealthiest household quintile and secondary or above education (b) among infants aged 12 months in Ethiopia, July 2020 to August 2021 (n= 1,850)
