# Supplementary material for: Geographical equity in Ethiopian infant feeding practices: a secondary analysis of a birth cohort study
Source: BMJ Open. 2025 Jan 2;15(1):e088762. doi: 10.1136/bmjopen-2024-088762 (PMC11749732; doi:10.1136/bmjopen-2024-088762)
Supplement: Supplementary file 3 [file bmjopen-2024-088762.reviewer_comments.pdf]

PEER REVIEW HISTORY

BMJ Open publishes all reviews undertaken for accepted manuscripts. Reviewers are asked to complete a checklist review form and are provided with free text boxes to elaborate on their assessment. These free text comments are reproduced below.

ARTICLE DETAILS

Title (Provisional)

Geographic equity in Ethiopian infant feeding practices: a secondary analysis of a birth cohort study

Authors

Tariku, Amare; Tariku, Amare; Persson, Lars; Schellenberg, Joanna; Marchant, Tanya; Berhanu, Della; Lemma, Seblewengel; Defar, Atkure; Zemedu, Theodros Getachew; Denu, Zewditu Abdissa; Delele, Tadesse Guadu; Shiferaw, Solomon; Zeleke, Girum Taye; Zelalem, Meseret; Gelaye, Kassahun Alemu

---

VERSION 1 - REVIEW

**Reviewer**	**1**
**Name**	**Lanigan, Julie**
**Affiliation**	**UCL GOS Institute of Child Health, Faculty of Pop Health Sciences**
**Date**	**20-Aug-2024**
**COI**	**I have no competing interests**

Congratulations on this well-conducted secondary analysis. The manuscript is well written; with an introduction that provides a clear justification for the research. Methods are fully explained and results are clearly presented.

I have some reservations regarding the study's limitations. I recommend authors acknowledge the limitations of reported data relating to breastfeeding practice in mothers. Although data collection was prospective this involved the administration of questionnaires to mothers. This method of data collection is subject to several sources of bias including recall and reporting bias in the case of the mothers. It should also be acknowledged that mothers may have been influenced by the involvement of researchers in the data collection (the Hawthorne or white coat effect). I am also concerned that a 1-day dietary recall is considered adequate to evaluate dietary diversity. Whilst it is acknowledged that dietary intake in infants in less diverse than in adults, there is still likely to be day-to-day variation. This would not be captured in a single dietary recall and this should be acknowledged with

future recommendations made to attempt more comprehensive dietary intake data collection in studies aiming to evaluate dietary diversity in the regions.

**Reviewer**	**2**
**Name**	**Herbison, Peter**
**Affiliation**	**University of Otago, Preventive and Social Medicine**
**Date**	**18-Sep-2024**
**COI**	**I have no competing interests**

I have no suggestions to make on this paper. I think it is fine as is.

VERSION 1 - AUTHOR RESPONSE

Reviewer 1: Comments to the Author: Congratulations on this well-conducted secondary analysis. The manuscript is well written; with an introduction that provides a clear justification for the research. Methods are fully explained and results are clearly presented.

Response: Many thanks.

1. I have some reservations regarding the study's limitations. I recommend authors acknowledge the limitations of reported data relating to breastfeeding practice in mothers. Although data collection was prospective this involved the administration of questionnaires to mothers. This method of data collection is subject to several sources of bias including recall and reporting bias in the case of the mothers. It should also be acknowledged that mothers may have been influenced by the involvement of researchers in the data collection (the Hawthorne or white coat effect).

   Response: Thank you. Yes, we prospectively measured infants' breastfeeding in three follow-up interviews, which is much better than the long recalls employed by the Ethiopia Demographic and health surveys. The local data collectors interviewed mothers which helps to minimize a 'White coat' bias. Discussions on the advantages of using of resident data collectors and prospective evaluation of breastfeeding is available at the second paragraph of the discussion section, Line 225-229.

2. I am also concerned that a 1-day dietary recall is considered adequate to evaluate dietary diversity. Whilst it is acknowledged that dietary intake in infants in less diverse than in adults, there is still likely to be day-to-day variation. This would not be captured in a single dietary recall and this should be acknowledged with future recommendations made to attempt more comprehensive dietary intake data collection in studies aiming to evaluate dietary diversity in the regions.

   Response: Thank you. It is correct that one 24-hour recall is insufficient to represent the individual dietary intake, but it has repeatedly been shown in different contexts to be a reasonably valid method on group level. Here, we report results for the group of one-year-old Ethiopian infants. We have discussed the limitation of using a single 24-hour recall in the second paragraph of the discussion section, Lines 233-235.

Reviewer 2: Comments to the Author: I have no suggestions to make on this paper. I think it is fine as is.

Response: Many thanks.
